# Two‐step workflow integrating automatic registration and manual refinement for the accurate alignment of serial histological sections in 3D reconstruction

**DOI:** 10.1111/joa.70203

**Published:** 2026-07-03

**Authors:** Satoru Muro, Takuya Ibara, Akimoto Nimura, Keiichi Akita

**Affiliations:** ^1^ Department of Clinical Anatomy, Graduate School of Medical and Dental Sciences Institute of Science Tokyo Tokyo Japan; ^2^ Department of Functional Joint Anatomy, Biomedical Engineering Laboratory, Institute of Industry Incubation Institute of Science Tokyo Tokyo Japan

**Keywords:** 3D reconstruction, image registration, serial histological sections

## Abstract

The accurate alignment of serial histological sections is essential for preserving anatomical continuity in 3D reconstruction. Although automated registration tools can efficiently correct global alignment errors, they often fail to resolve local misalignments caused by sectioning artifacts, tissue deformation, staining variability, or missing slices. Thus, we propose a practical two‐step registration workflow that uses MultiStackReg (an ImageJ/Fiji plugin) for automatic alignment and AlignRef (a standalone application for interactive adjustment) for manual refinement. In the first step, MultiStackReg performs global registration by using rigid body transformations. In the second step, AlignRef corrects residual misalignments through semi‐transparent overlay visualization, keyboard‐based translation and rotation, and batch propagation of recorded transformations across selected slice ranges. We applied our workflow to 135 serial sections of a Carnegie Stage 15 embryo from the Virtual Human Embryo dataset that were stained with hematoxylin and eosin. MultiStackReg resolved most global inconsistencies, whereas AlignRef enabled the precise adjustment of subtle local deviations, particularly in curved structures such as neural tubes and limb buds. After automatic registration with MultiStackReg, the subsequent manual refinement step using AlignRef was completed in approximately 30 min and produced a suitable stack for 3D reconstruction. This two‐step workflow balances automation with expert‐guided correction and provides an accessible, reproducible, and anatomically precise method for the serial section alignment of morphological and developmental anatomy.

## Graphical abstract

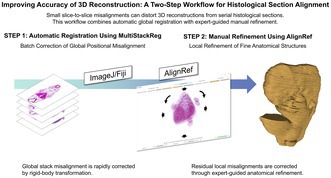

## 1 INTRODUCTION

Accurate section registration is required to preserve anatomical continuity in 3D reconstructions derived from serial histological sections (Muro, Chang, et al., 2025; Muro, Shoji, et al., 2025). In morphological and developmental anatomy, even small positional inconsistencies between adjacent slices can lead to geometric distortions in reconstructed volumes. Such distortions are commonly corrected by using automated registration methods; however, these methods often fail to fully resolve local misalignments caused by tissue deformation, staining irregularities, or missing sections.

**FIGURE 1:**
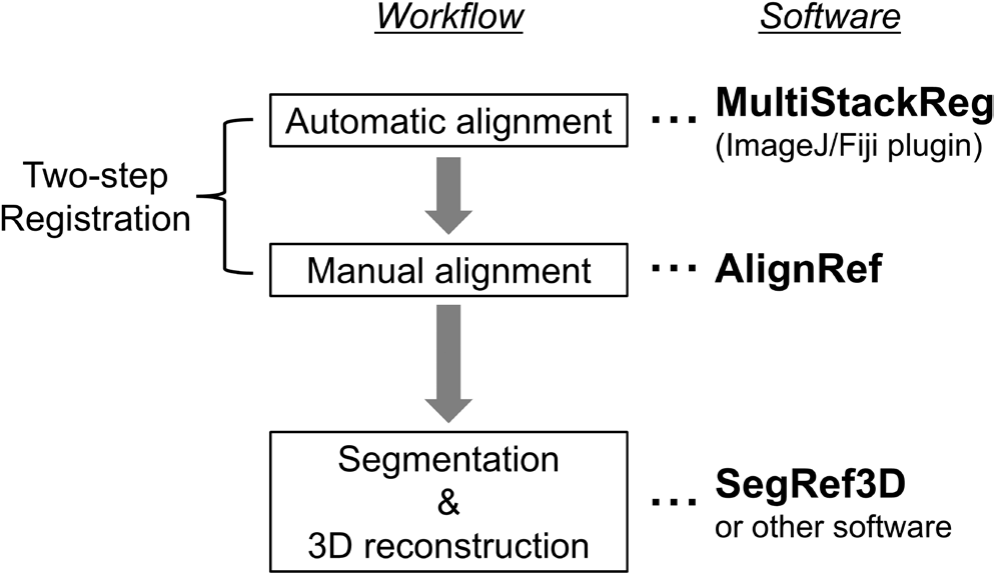
Overview of the two‐step workflow for the registration of serial histological sections. To correct residual misalignments prior to segmentation and 3D reconstruction, automatic alignment is performed using MultiStackReg (an ImageJ/Fiji plugin) followed by manual fine adjustment using AlignRef.

In this study, we present a practical two‐step workflow that combines MultiStackReg (an ImageJ/Fiji plugin), an automatic registration method, with AlignRef, a standalone manual refinement tool that we have developed (Figure 1). This combination achieved both efficiency and anatomical precision in the alignment of serial histological sections prior to segmentation and 3D reconstruction.

## 2 METHODS

The proposed two‐step registration workflow, which uses automatic alignment using MultiStackReg followed by manual refinement using AlignRef, was applied to serial sections from the Virtual Human Embryo dataset (Carnegie Stage 15) that were stained with hematoxylin and eosin. The dataset comprised 135 serial sections with a 60‐μm interslice interval.

### 2.1 Automatic registration using MultiStackReg

Automatic alignment was first performed using MultiStackReg (https://github.com/miura/MultiStackRegistration) (Thévenaz et al., 1998), a plugin for ImageJ/Fiji. This plugin is based on TurboReg and provides multiple transformation modes, namely, translation, rigid body, scaled rotation, and affine, thus allowing users to select the appropriate level of geometric correction. The rigid body mode, which includes translation and rotation, was found to be suitable for serial histological sections wherein global deformation is minimal.

#### 2.1.1 Installation and setup

MultiStackReg and its dependency TurboReg can be installed using the Fiji Update Manager as follows: launch Fiji, select the Help menu, click Update, open Manage Update Site, search for “multistackreg” and “BIG‐EPFL,” enable both plugins, click on Apply Changes, and restart Fiji to complete the installation. Once installed, MultiStackReg can be accessed under Registration in the Plugins menu.

#### 2.1.2 Procedure for automatic registration

All serial section images were organized in a single folder with numerically sorted filenames (e.g., image0001–image0135). The sequence was imported into Fiji via File > Import > Image Sequence…, thus ensuring that the filenames were numerically validated. Given that MultiStackReg cannot directly process color images, the RGB channels were split using Image > Color > Split Channels, thus producing three grayscale stacks labeled (red), (green), and (blue).

Registration was executed on a one‐channel stack—chosen arbitrarily from the split RGB channels (e.g., the first channel, which ImageJ labels as “blue”)—by selecting Plugins > Registration > MultiStackReg, choosing the desired stack, and selecting Action 1: Align and Transformation: Rigid Body. The Save Transformation File option was checked to store the computed transformation matrix. After execution, the resulting transformation file (e.g., TransformationMatrices.txt) was applied to the remaining two channel stacks (green and red) using Action 1: Load Transformation File.

Finally, the aligned color channels were recombined using Image > Color > Merge Channels. In the Merge Channels dialog, the “Create composite” checkbox was left unchecked so that the channels were merged into a standard RGB image rather than a composite image. The recombined RGB image sequence was then saved via File > Save As > Image Sequence. This process yielded an automatically registered stack of color images.

#### 2.1.3 Limitations of the automatic approach

Although MultiStackReg provides fast and robust global registration, it cannot always correct for local distortions or discontinuities in histological sections owing to manual sectioning artifacts or variable tissue thickness. Consequently, small positional deviations often persist, particularly in delicate structures such as nerves or fine connective tissues. These residual errors can interfere with accurate segmentation and 3D reconstruction, thus necessitating subsequent manual adjustments.

### 2.2 Manual alignment using AlignRef

To correct residual misalignments after automatic registration, we developed and utilized AlignRef (https://github.com/SatoruMuro/AlignRef), a lightweight, standalone desktop application for the manual alignment of serial images. The software operates entirely offline and provides a graphical user interface implemented in Python 3.11 and PyQt6. The compiled executable requires no installation and currently runs on Windows 10/11; however, the program is fully compatible with macOS and Linux when executed using the Python source code.

#### 2.2.1 Overview and key features

**FIGURE 2:**
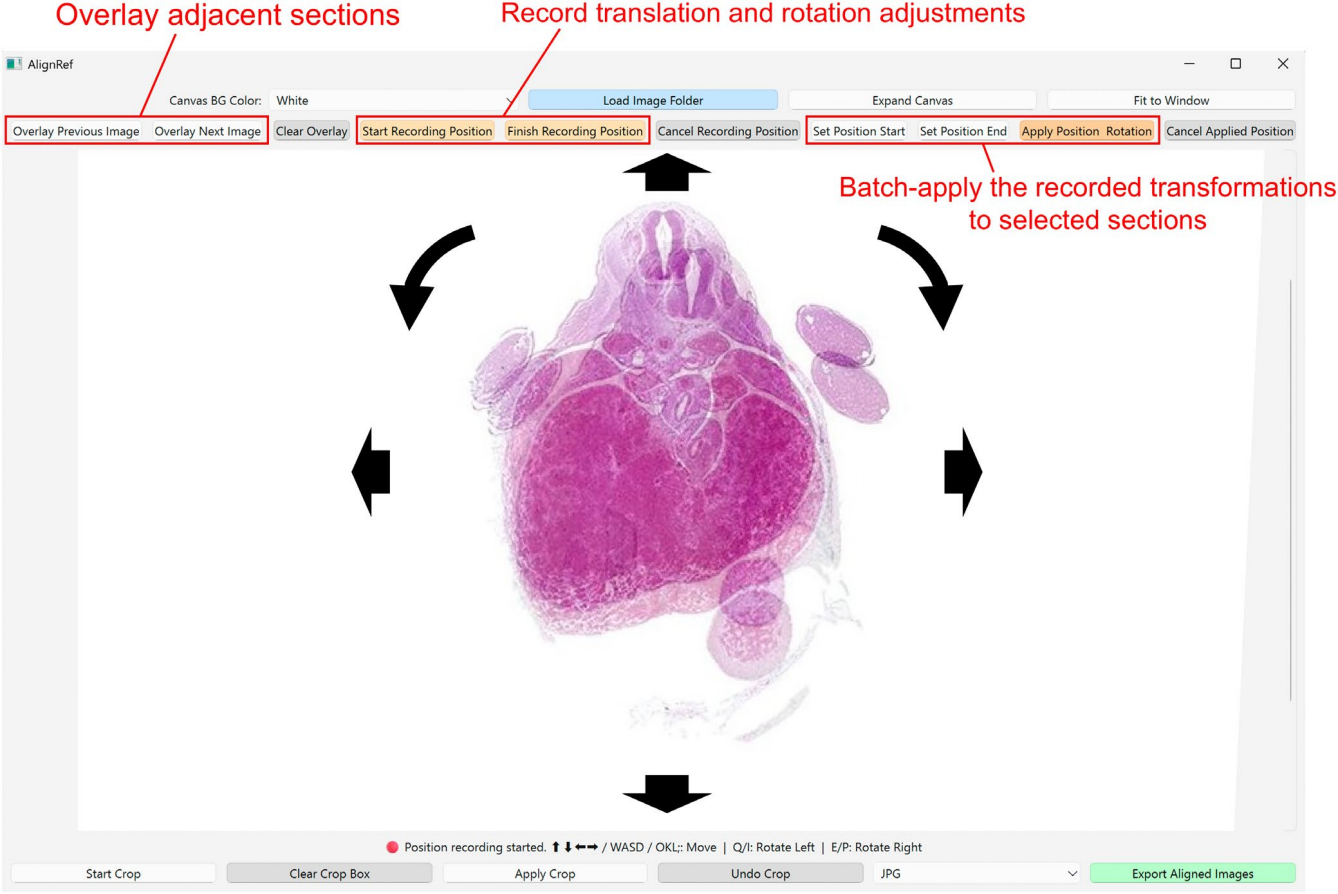
Graphical user interface of AlignRef for multi‐section alignment. The AlignRef interface has a semi‐transparent overlay of adjacent sections and uses a keyboard‐based fine‐adjustment workflow. Users can record translation and rotation adjustments by using the Start/Finish Recording Position and then apply the recorded transformations to any selected range of sections by using Set Position Start/End followed by Apply Position Rotation. Section images can be switched using keyboard shortcuts during the alignment process.

AlignRef was designed with simplicity and reproducibility (Figure 2). Its key features are as follows:

1. Unified canvas: automatically resizes all images to a consistent canvas size for smooth navigation and manipulation.
2. Overlay visualization: a semi‐transparent display of the previous or next image to ensure visual continuity across adjacent sections.
3. Keyboard‐based adjustments: translation (W, A, S, D, or arrow keys) and rotation (Q/E keys); users can interactively fine‐tune the position and rotation of each image.
4. Batch transformation: once an optimal alignment is achieved for a representative section, the same translation and rotation can be propagated to a defined range of slices.
5. Cropping and export: cropping boxes can be applied uniformly to all aligned images, and the outputs can be exported in multiple formats (JPG, PNG, TIFF, BMP, and DICOM). The exported data are automatically stored in a new folder labeled with the original dataset name and timestamp (e.g., dataset_aligned_20251021_1030).

The maximum processable dataset size is not defined by a fixed software limit, but depends primarily on image resolution, the number of sections, and available system memory. Because AlignRef retains image data in memory for interactive display and manual refinement, we recommend resizing section images so that the longer side is approximately 1000 pixels or less for routine use. Under this recommended condition, practical memory requirements for representative dataset sizes are summarized in Table S1.

#### 2.2.2 Workflow and operation

The typical workflow when using AlignRef includes the following:

1. Load all serial images through Load Image Folder.
2. Optionally add a 100‐px canvas margin by using Expand Canvas for alignment convenience.
3. Use Overlay Previous/Next Image to visually confirm continuity between adjacent sections.
4. Press Start Recording Position on the section to be adjusted, and then translate or rotate the displayed image using the keyboard while comparing it with the semi‐transparent overlay. Section images can be switched using PageDown/F/J for the next image and PageUp/R/U for the previous image.
5. Once the optimal alignment has been determined, press Finish Recording Position to record the translation and rotation values.
6. Specify the frame range to which the recorded transformation should be applied using Set Position Start/End.
7. Apply the recorded transformation across the selected range using Apply Position Rotation.
8. Optionally crop by using Start Crop and Apply Crop, and then export the aligned images.

All operations are interactive and provide immediate onscreen updates. AlignRef does not require coding or scripting knowledge, thereby enabling users working with anatomical or morphological images to perform accurate manual corrections with minimal technical effort.

### 2.3 Ethics statement and patient consent

This study involved the development of a research tool and did not involve human participants, identifiable data, or patient materials. Therefore, ethical approval and patient consent were not required.

## 3 RESULTS

MultiStackReg successfully aligned the majority of sections automatically; however, subtle local misalignments remained. These residual deviations were interactively corrected by using AlignRef, particularly around curved regions such as the neural tube and limb buds. Furthermore, semi‐transparent overlay visualization facilitated the precise matching between adjacent sections, thus ensuring smooth anatomical continuity. After automatic registration with MultiStackReg, the subsequent manual refinement step using AlignRef took approximately 30 min on a standard Windows 10 computer with an Intel Core i7 CPU and 16 GB RAM. Compared with manual‐only alignment, the proposed two‐step workflow substantially reduced the amount of manual adjustment required because most global positional inconsistencies were corrected automatically by MultiStackReg. Compared with automated registration alone, the addition of AlignRef improved anatomical continuity by allowing residual local misalignments to be corrected through direct visual comparison of adjacent sections. Thus, the proposed workflow achieved a practical balance between processing efficiency and anatomically guided accuracy.

**FIGURE 3:**
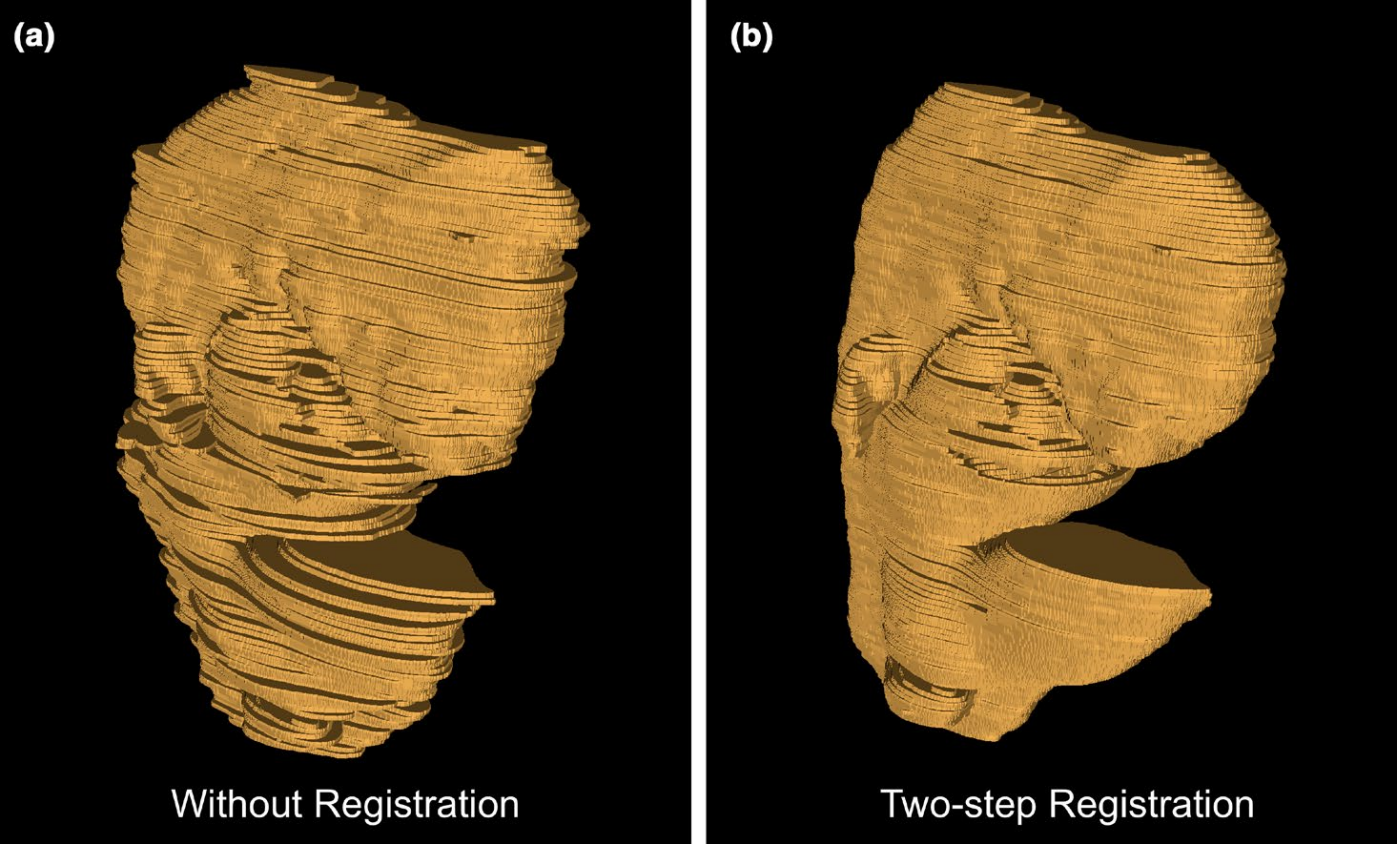
Example of histological 3D reconstruction before and after two‐step registration workflow. Three‐dimensional reconstructions generated from serial histological sections of a Carnegie Stage 15 embryo. (a) Reconstruction before two‐step registration workflow showing a marked slice‐to‐slice misalignment. (b) Reconstruction after the two‐step registration workflow demonstrating improved alignment and structural continuity.

The resulting aligned images were exported as JPG files and then used for 3D reconstruction in SegRef3D (ver. 1.1.0; https://github.com/SatoruMuro/SAM2GUIfor3Drecon) (Muro, Ibara, et al., 2026). This demonstrated the compatibility of the proposed workflow with existing segmentation and visualization pipelines (Figure 3).

## 4 DISCUSSION

The use of MultiStackReg and AlignRef provides a practical and reproducible solution for serial section alignment in morphological studies. Although automated registration significantly accelerates processing, manual refinement remains essential for achieving anatomical accuracy, particularly in heterogeneous tissue staining, section deformation, or partial section loss. AlignRef complements automated tools by offering human‐in‐the‐loop corrections, thereby allowing users to apply expert anatomical judgments within a standardized software interface that provides consistent overlay visualization, keyboard‐based translation and rotation, and reproducible batch application of recorded transformations. Its lightweight implementation, open‐source availability, cross‐platform compatibility, and offline operability enable its use in laboratory or educational settings without specialized hardware.

The proposed workflow reflects a realistic balance between automation and expert intervention. In educational contexts, students can visually explore the continuity of structures across slices by using AlignRef, thereby enhancing their 3D understanding of anatomical morphology. In addition to conventional histological serial sections, the same two‐step registration workflow is also applicable to serial images obtained using correlative microscopy and block‐face imaging (CoMBI) (Ishii et al., 2021). CoMBI acquires high‐resolution block‐face images during serial sectioning. Similar to histological sections, the generated datasets require accurate registration to maintain spatial continuity before 3D reconstruction (Muro, Ochi, et al., 2026). Considering that both MultiStackReg and AlignRef operate on standard image sequences independent of the staining or imaging modality, the same alignment procedure can be directly applied to the CoMBI datasets for correlative 3D morphological analysis.

Future enhancements of AlignRef may focus on improving user‐interface efficiency and expanding its ability to handle larger datasets. As automated registration tools continue to advance, AlignRef is expected to develop further as a practical companion tool for making precise anatomically guided refinements that remain essential even after automated alignment. This complementary role reinforces their utility in real‐world workflows for serial section‐based reconstructions.

## 5 CONCLUSION

A workflow that uses MultiStackReg for automatic registration and AlignRef for manual refinement provides a reliable and accessible workflow for aligning serial histological sections. This method ensures efficiency and anatomical precision, thus making it suitable for research applications involving 3D reconstruction and segmentation.

AlignRef is freely available as a standalone executable on GitHub (https://github.com/SatoruMuro/AlignRef).

## AUTHOR CONTRIBUTIONS

Satoru Muro conceived the study, developed the methodology and software, performed the data analysis, and drafted the manuscript. Takuya Ibara supported the software development and validation. Akimoto Nimura provided critical feedback on the manuscript. Keiichi Akita supervised the study. All authors have reviewed the manuscript and approved the final version for publication.

## Supporting information


Table S1.
**Table S2**.

## Data Availability

The data that support the findings of this study are openly available in AlignRef at https://github.com/SatoruMuro/AlignRef.
