# Supplementary material for: Two‐step workflow integrating automatic registration and manual refinement for the accurate alignment of serial histological sections in 3D reconstruction
Source: J Anat. 2026 Jul 3:10.1111/joa.70203. Online ahead of print. doi: 10.1111/joa.70203 (PMC13398511; doi:10.1111/joa.70203)
Supplement: Supplementary file 1 — Table S1. Table S2. [file JOA-9999-0-s001.docx]

**Supplementary Table S1. Practical guide to dataset size and estimated memory usage in AlignRef**

| Number of sections | Recommended image size | Approximate raw RGB data size | Estimated practical memory usage |
| --- | --- | --- | --- |
| 100 | Long side ≤ 1000 px | ~0.3 GB | ~1–2 GB |
| 300 | Long side ≤ 1000 px | ~0.9 GB | ~3–6 GB |
| 500 | Long side ≤ 1000 px | ~1.5 GB | ~5–10 GB |
| 1000 | Long side ≤ 1000 px | ~3.0 GB | ~10–20 GB |

Values are approximate estimates based on RGB images resized so that the longer side is approximately 1000 pixels or less. Raw RGB data size was calculated assuming an image size of approximately 1000 × 1000 pixels and 3 bytes per pixel. Practical memory usage is expected to be higher because AlignRef retains image data in memory for interactive display and manual refinement, and additional memory may be required for cached images, display objects, and temporary arrays. Actual memory usage may vary depending on the operating system, image format, and operations performed.

**Supplementary Table S2. Qualitative comparison of serial-section alignment workflows**

| Workflow | Accuracy | Processing time | Practical limitations |
| --- | --- | --- | --- |
| Manual-only alignment | Potentially high, but highly dependent on operator experience | Long, because each section must be adjusted individually | Time-consuming and less reproducible |
| Automated registration alone | Efficient for global alignment | Short | Residual local misalignments may remain, especially in curved or delicate structures |
| Proposed two-step workflow | High practical accuracy through automated global alignment and expert-guided local refinement | Approximately 30 min for manual refinement after automatic registration in the present 135-section dataset | Requires visual inspection and manual correction of residual misalignments |

This table provides a qualitative comparison of representative alignment workflows based on workflow characteristics and the present application to 135 serial histological sections. Processing time may vary depending on image resolution, number of sections, computer specifications, and the extent of residual misalignment requiring manual correction.
